# Mechanisms of postischemic cardiac death and protection following myocardial injury

**DOI:** 10.1172/JCI184134

**Published:** 2025-01-02

**Authors:** Yusuf Mastoor, Elizabeth Murphy, Barbara Roman

**Affiliations:** Laboratory of Cardiac Physiology, National Heart, Lung, and Blood Institute, NIH, Bethesda, Maryland, USA.

## Abstract

Acute myocardial infarction (MI) is a leading cause of death worldwide. Although with current treatment, acute mortality from MI is low, the damage and remodeling associated with MI are responsible for subsequent heart failure. Reducing cell death associated with acute MI would decrease the mortality associated with heart failure. Despite considerable study, the precise mechanism by which ischemia and reperfusion (I/R) trigger cell death is still not fully understood. In this Review, we summarize the changes that occur during I/R injury, with emphasis on those that might initiate cell death, such as calcium overload and oxidative stress. We review cell-death pathways and pathway crosstalk and discuss cardioprotective approaches in order to provide insight into mechanisms that could be targeted with therapeutic interventions. Finally, we review cardioprotective clinical trials, with a focus on possible reasons why they were not successful. Cardioprotection has largely focused on inhibiting a single cell-death pathway or one death-trigger mechanism (calcium or ROS). In treatment of other diseases, such as cancer, the benefit of targeting multiple pathways with a “drug cocktail” approach has been demonstrated. Given the crosstalk between cell-death pathways, targeting multiple cardiac death mechanisms should be considered.

## Overview of ischemia/reperfusion injury

**Figure 1:**
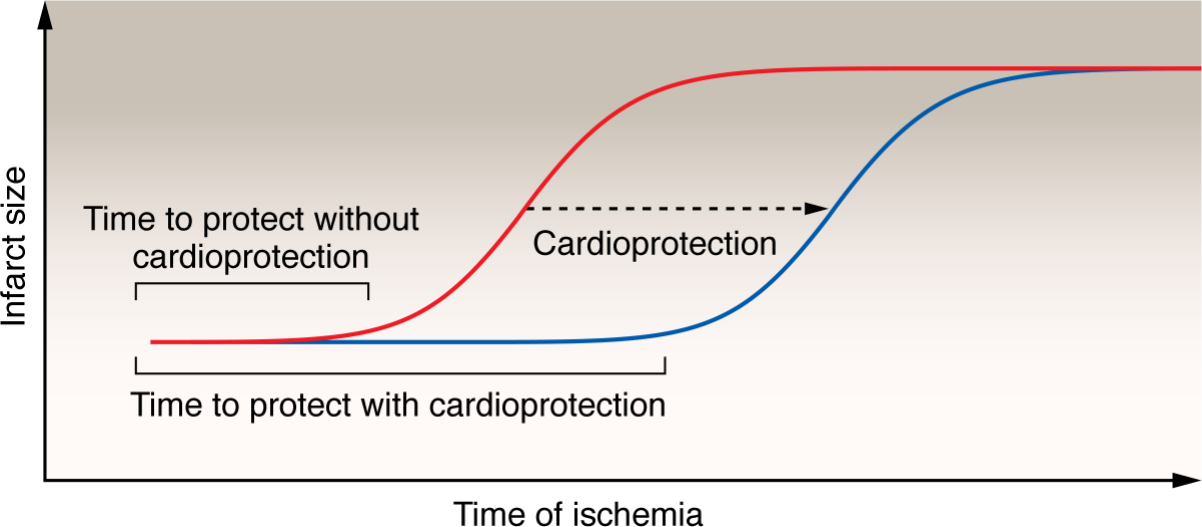
Theoretical relationship between ischemia and infarct size. The figure illustrates the general relationship between the time of ischemia and infarct size (red). At the start of and during early ischemia, there is little to no cell death if timely reperfusion therapy is administered. As the time of ischemia increases, irreversible myocardial damage occurs until most of the heart is damaged. Applying a cardioprotective strategy along with reperfusion therapy (including PCI or thrombolysis) extends the early window during which most of the undamaged heart tissue can be saved. This shifts the curve to the right (blue line), representing more time to protect. However, it is unlikely that any cardioprotective strategy will completely prevent an infarct if cardioprotection is applied too late.

Ischemic heart disease due to acute myocardial infarction (MI) is the leading cause of death in the world. As cardiomyocytes rely on oxygen to meet the heart’s metabolic demands by generating ATP via oxidative phosphorylation, a lack of oxygen and blood flow — as happens during ischemia — impairs cardiomyocyte function and ultimately leads to cell death. It is therefore essential to restore blood flow and oxygen to the myocardium soon after the ischemic event. It should be emphasized that there is a somewhat narrow window in which to implement cardioprotective interventions (Figure 1). If the heart is reperfused during early ischemia, there is no cell death. As the time of ischemia increases, the percentage of the myocardium that dies gradually increases until essentially all heart tissue is dead. The precise timing depends on the species and the model of ischemia. Cardioprotective strategies shift the curve to the right, such that the level of cell death is lower at a given time of ischemia. However, it is unlikely that any cardioprotective drug or strategy can protect a cardiomyocyte from death if ischemia is prolonged. In this Review, we focus on cardioprotective interventions that reduce acute cell death. Due to space limitations, we do not discuss protective strategies such as SGLT2 inhibitors and other promising approaches that modify remodeling and heart failure.

**Figure 2:**
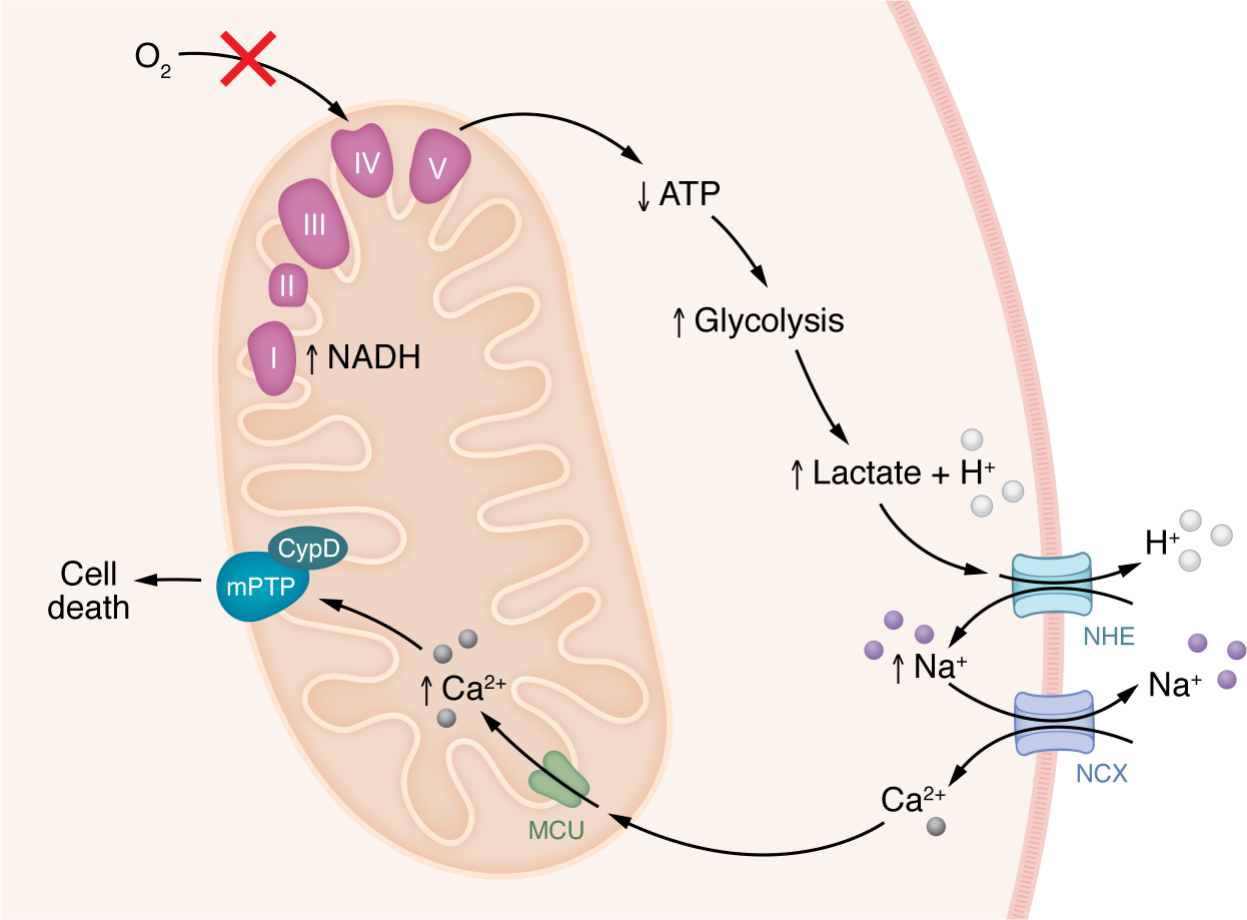
Molecular changes in the cell during cardiac ischemia. Cardiac ischemia and the resultant lack of oxygen lead to cessation of aerobic metabolism, transition to anaerobic metabolism, accumulation of glycolytic byproducts such as succinate and lactate, a decrease in intracellular pH, and increases in cytosolic Na^+^ and Ca^2+^ (9). Without oxygen to accept electrons from complex IV, the ETC is inhibited, and NADH and FADH_2_ accumulate. ATP production via complex V (also known as ATP synthase) stops, and the heart must rely on glycolysis as the predominant pathway for ATP generation. During ischemia, approximately 50% of the glycolytically generated ATP is consumed by the reverse mode of the F_1_F_0_-ATP synthase and used to maintain mitochondrial membrane potential (Δψ) (10, 199, 200). In the cytosol, glucose is metabolized to pyruvate and subsequently lactate, resulting in acidosis of the cytosol due to retention of protons from degradation of glycolytically generated ATP (9, 198). The increase in cytosolic proton concentration stimulates H^+^ efflux via the Na^+^/H^+^ exchanger (NHE) (120). Na^+^ that enters is not extruded due to dysfunction of the Na^+^/K^+^ pump. The increase in cytosolic Na^+^ stimulates plasma membrane Na^+^/Ca^2+^ exchanger (NCX), leading to an increase in cytosolic Ca^2+^ (120, 134, 201). An increase in mitochondrial Ca^2+^ has also been recently shown to occur during ischemia and is thought to lead to cell death through opening of the mitochondrial permeability transition pore (mPTP) (128, 129, 134). Opening of the pore has been shown to be regulated by cyclophilin D (CypD).

**Figure 3:**
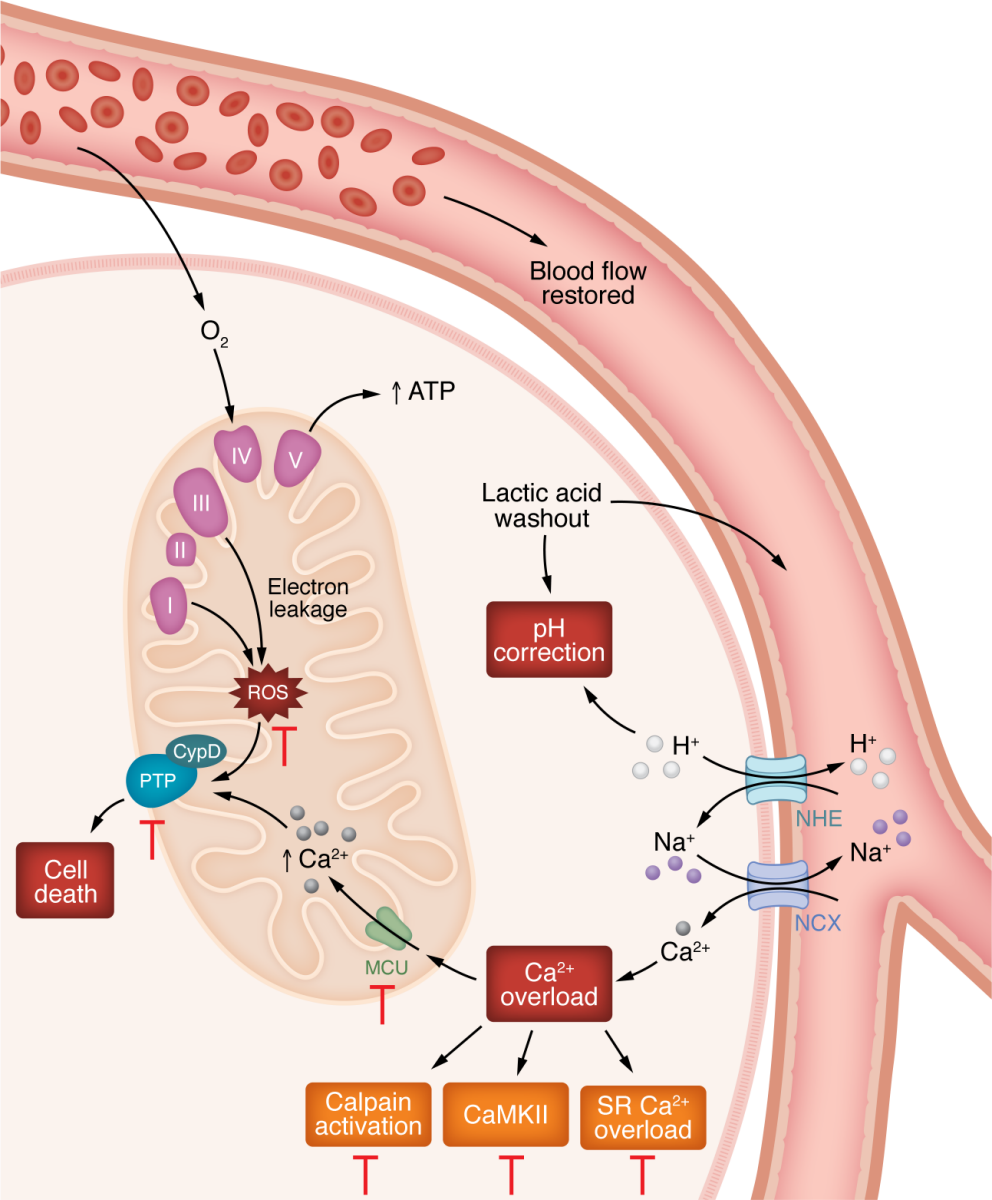
Molecular changes in the cell after reperfusion. The return of oxygen during reperfusion generates ROS, primarily by mitochondria (10, 11). Damage to the ETC during ischemia leads to increased ROS production on reperfusion. Complex I and complex III are the primary sites of ROS production in the mitochondria (11, 160, 202), but other sites can also contribute (203). The increase in succinate that occurs during ischemia can lead to RET through generation of ROS by complex I. Extracellular pH is rapidly restored, which promotes extrusion of intracellular H^+^ via NHE, leading to a transient increase in intracellular Na^+^. As ATP is restored, the Na^+^-K^+^ ATPase becomes active and helps to extrude intracellular Na^+^. Depending on the relative timing of ATP restoration, a sustained increase in cytosolic Na^+^ can stimulate NCX, leading to a further increase in cytosolic Ca^2+^ during early reperfusion. ROS can also lead to damage of intracellular proteins such as SERCA and RyR2, leading to altered SR Ca^2+^ homeostasis. Together, these can lead to greater Ca^2+^ accumulation in the cytosol and exacerbate reperfusion injury. Any increase in cytosolic Ca^2+^ present at the start of reperfusion would lead to an increase in mitochondrial Ca^2+^ accumulation via MCU when the Δψ is restored on reperfusion (204). This further increase in mitochondrial Ca^2+^ on reperfusion depends on how fast Δψ is restored relative to how quickly cytosolic Ca^2+^ returns to baseline. Ca^2+^ overload in the mitochondria is thought to prime the mPTP to open on reperfusion when pH is restored. It is widely cited that mPTP activation is inhibited by the acidic pH induced by ischemia (205, 206) and that upon reperfusion, intracellular and extracellular pH are rapidly corrected, allowing for mPTP opening. However, inhibition of mPTP by acidic pH only occurs in de-energized mitochondria. In energized mitochondria, low pH actually enhances mPTP opening (207). ROS is another activator of mPTP, and it is likely that the increase in ROS that occurs during reperfusion synergizes with the increase in mitochondrial Ca^2+^ (which may already be there during ischemia) to activate mPTP on reperfusion.

During ischemia, inhibition of aerobic metabolism, inhibition of oxidative metabolism, and alterations in ionic homeostasis occur (Figure 2), and without reperfusion there is no cell survival. Upon reperfusion, oxygen and other substrates required for aerobic respiration are restored (Figure 3), but the reintroduction of oxygen contributes to an increase in production of ROS (1, 2). Reperfusion can also lead to a further accumulation of intracellular Ca^2+^ in or entry of Ca^2+^ into mitochondria (3, 4) and opening of the mitochondrial permeability transition pore (mPTP) (5, 6). While it is difficult to determine how much cardiac damage results from ischemia versus reperfusion, some have proposed that reperfusion injury accounts for around 50% of the total infarct (7).

## Cell-death pathways

Understanding how regulated cell-death pathways contribute to I/R injury could provide new insights for the development of therapeutic strategies to attenuate acute myocardial damage. Although many cell-death pathways have been defined, not all have been shown to play a role in myocyte damage during I/R injury (8). In this section, we highlight the major regulated cell-death pathways involved in I/R injury and how they may interconnect. A few caveats should be considered regarding the conclusion that a pathway is involved in I/R injury. A model with knockout or overexpression of a protein in a death-signaling pathway is commonly used to demonstrate the involvement of that pathway in I/R injury. However, in many of these studies, the knockout or overexpression occurs at birth or before, and compensatory changes might be responsible for the altered death response. It is also becoming clear that there is considerable overlap among the cell-death pathways and that the distinction between them may be somewhat artificial. For example, loss of BCL2 has been shown to reduce both apoptosis and necrosis. It is also worth noting that I/R injury almost certainly involves rupture of the plasma membrane. Therefore, it is important to better understand how the different cell-death mechanisms ultimately lead to plasma membrane rupture in the setting of I/R.

### Necrosis

Traditionally, necrosis is characterized by cellular swelling, organelle dysfunction, and plasma membrane rupture, leading to the release of cytosolic components resulting in an inflammatory response (9). Necrosis is thought to lead to the increased release of the intracellular components troponin and creatine kinase that occurs during early reperfusion.

Although necrosis was originally thought to be an unregulated cell death pathway, it now appears that necrosis can be regulated. Necrosis is proposed to lead to opening of the mPTP. In the context of I/R injury, mPTP opening is primarily triggered by the Ca^2+^ overload and increased ROS production that occur at the start of reperfusion (10). Once the mPTP is opened, the proton gradient across the inner mitochondrial membrane (IMM), which is responsible for mitochondrial ATP production, is dissipated (5). Opening of the mPTP exacerbates ROS generation, causing oxidative damage to cellular components (8, 11). In addition, mPTP opening permeabilizes the IMM to molecules less than 1.5 kDa, which leads to mitochondrial swelling from the influx of solutes and water (12, 13). The loss of ATP and increased ROS are thought to initiate cell death. However, how these changes lead to plasma membrane rupture needs additional study.

A number of mitochondrial proteins have been implicated in mPTP opening, as reviewed previously (4). F_1_-F_0_-ATPase (F_1_-ATPase) and adenine nucleotide translocase (ANT) have both been proposed to function as the mPTP (14–16). One study found that dimers of the F_1_-ATPase can form the pore-forming unit of the mPTP, whereas another study reported that delipidation of the c-ring of the F_1_-ATPase can form the mPTP (14, 17). However, studies from Walker’s group have disputed a role for the F_1_-ATPase in the mPTP (18, 19). As discussed below, cyclosporin A (CsA) binds to a matrix protein, cyclophilin D (CypD), and desensitizes the mPTP (20, 21); Walker’s group has shown that in the presence of CsA, an mPTP-like swelling can still occur in cells lacking the c-ring and other components of the F_1_-ATPase that are proposed to be involved in mPTP formation. As an explanation for these discrepancies, it has been suggested that there are multiple pores inhibited by CsA (15, 16, 22). ANT was originally proposed as the mPTP based on data showing that ANT interacts with CypD and adenine nucleotides. When ANT was locked in the matrix-facing conformation by use of bongkrekic acid, mPTP was inhibited, whereas ANT locked in the cytosolic-facing conformation by use of carboxyatractyloside led to mPTP activation (13). However, the hypothesis was discarded when Wallace’s group reported that CsA-inhibitable mitochondrial swelling still occurs with deletion of ANT1 and -2, although more Ca^2+^ is required for activation (23). Recently, Karch et al. (24) showed that deleting ANT1, -2, and -4 from mitochondria desensitized mitochondria to Ca^2+^-induced mPTP opening, and further addition of CsA completely inhibited mPTP opening. They proposed two distinct mPTPs: ANT and a second pore. Both components may be activated by CypD, although the non-ANT component has a strict requirement for CypD.

While the exact structure and molecular identity of the mPTP are debated, CypD — a peptidyl-prolyl *cis-trans* isomerase located within the mitochondrial matrix — has been identified as a regulator of pore opening in response to Ca^2+^. CsA binds to CypD and inhibits its isomerase activity. CsA has been shown in animal studies to reduce mPTP pore opening during I/R (25–28) and to reduce cell death (29, 30). When the gene encoding CypD, *Ppif*, was deleted in mice, Ca^2+^-induced opening of the mPTP as well as necrotic cell death following in vivo I/R were both inhibited (31, 32).

To address the role of isomerase activity in CypD-mediated mPTP activation, a mutant of CypD lacking prolyl isomerase activity (CypD R96G) was created (33). Mitochondria from the CypD R96G mouse had reduced Ca^2+^-activated mPTP opening, and addition of CsA provided no additional protection. Surprisingly, hearts from CypD R96G mutant mice subjected to 20 minutes of ischemia and 90 minutes of reperfusion showed no protection against I/R injury. These data suggest that CypD, independent of its isomerase activity, plays a role in the pathogenesis of I/R injury. These results align with the theory that there are two mPTPs (33): one of the mPTP conformations is activated by CypD isomerase activity, whereas the other is activated by CypD but does not require its isomerase activity. Furthermore, the mPTP, which contributes to cell death in I/R, is enhanced by a mutant CypD devoid of isomerase activity.

### Necroptosis

Necroptosis, which has been reviewed in detail elsewhere (8), is a regulated form of necrosis that can be triggered by plasma-membrane death receptors. In the classic necroptotic pathway, the binding of a ligand to its death receptor leads to a cascade of signals that activate receptor-interacting protein kinase 1 (RIPK1), which phosphorylates RIPK3. RIPK3 then phosphorylates the pseudokinase mixed-lineage kinase–like domain (MLKL) (34, 35). MLKL oligomerizes and permeabilizes the plasma membrane and targets mitochondrial metabolic enzymes to induce cell death and increase ROS production (36, 37). This plasma membrane permeabilization leads to an influx of ions, resulting in necrotic membrane rupture due to increased osmotic pressure (38).

Necroptosis was initially implicated in cell death during I/R when an allosteric inhibitor of RIPK1, necrostatin-1, was found to reduce infarct size when administered upon reperfusion in animal models (39–41). This was also associated with a lower level of necrotic cell death and decreased phosphorylation of RIPK1 and RIPK3. It was also shown that the major mediators of necroptosis, RIPK1, RIPK3, and MLKL, were all increased in an in vivo mouse model of I/R (42). Furthermore, when RIPK3 was completely knocked out in mice, following I/R, the infarct size was reduced, cardiac function was improved, and ROS production was decreased (43). However, siRNA silencing of MLKL, the classical effector of RIPK3, concurrent with RIPK3 overexpression did not completely block necroptosis in cardiomyocytes, indicating that other effectors of RIPK3 may also be important in the context of I/R injury (43). Interestingly, it was found that overexpression of RIPK3 triggered necroptosis through Ca^2+^/calmodulin-dependent protein kinase IIδ (CaMKIIδ), a regulator of mPTP and another effector of RIPK3, independently of RIPK1 or MLKL (43, 44). In addition, inhibiting CaMKIIδ with RIPK3 overexpression decreased cardiomyocyte death in response to I/R.

There appears to be crosstalk between mPTP opening and necroptosis. Necroptosis has been reported to activate the mPTP (45). Linkermann et al. (46) showed that protection provided by CsA and necrostatin is additive. Interestingly, hearts with germline deletion of the mitochondrial Ca^2+^ uniporter (MCU) were not protected from I/R injury, and although CsA and necrostatin each reduced I/R injury in WT littermates, neither showed any protection in germline MCU-KO hearts. These data suggest that the rewiring of cell-death mechanisms that appears to occur in the germline MCU-KO heart interferes with protection by both CsA and necrostatin, consistent with some overlap in these pathways.

### Ferroptosis

Recent experiments demonstrate that ferroptosis, a newly described cell-death pathway, is an important contributor to I/R injury (47). Ferroptosis is an iron-dependent programmed cell death pathway (48–50) that morphologically differs from necrosis and apoptosis, presenting with dense, compact mitochondria with loss of cristae (48). It is characterized by lipid peroxidation and oxidative damage to cellular membranes (8, 51). The Fenton reaction generates ferric iron (Fe^3+^), leading to increased ROS levels and activation of lipoxygenases, which damage cellular membranes, particularly those containing phosphatidyl ethanolamine–containing polyunsaturated fatty acids (PUFAs) (52). It has been demonstrated that molecules such as deferoxamine and dexrazoxane, which chelate iron (53), or those that oppose lipid peroxidation, such as ferrostatin-1, liproxstatin-1, and vitamin E (48, 54), can prevent lipid peroxidation and block ferroptosis. Interestingly, a recent study demonstrated that the effect of ferrostatin and CsA treatment was additive in reducing infarct size (55).

Recent studies have demonstrated that the MCU can transport iron into mitochondria and provided data suggesting that cardiac deficiency of MCU can be a mechanism for reducing ferroptosis and cardiac damage (56). Another therapeutic approach for preventing ferroptosis in myocardial I/R injury is activation of PPARα (57). The PPARα/14-3-3η pathway has been shown to protect cardiomyocytes from ferroptosis and mitochondrial injury (57). Additionally, Mito-TEMPO, a mitochondria-targeted antioxidant, has been shown to prevent ferroptosis caused by doxorubicin treatment in the heart (58), suggesting its potential as a cardioprotective agent.

### Apoptosis

Apoptosis is characterized by the involvement of initiator and effector proteins, leading to changes in both the nucleus and cytoplasm (59). The details of this pathway are reviewed elsewhere (8). Data suggest that the pathway or elements of the pathway can also play a role in I/R. Numerous experiments involving genetic and pharmacological manipulation of apoptosis proteins have demonstrated a reduction in infarct size in animals subjected to I/R (60–66).

In the canonical intrinsic pathway, apoptotic signaling is triggered by the mitochondria and is regulated by BCL-2 family proteins (67). This mechanism involves an increase in the permeability of the outer mitochondrial membrane (OMM), leading to the release of proapoptotic factors, including cytochrome *c*, into the cytoplasm. In the presence of ATP, cytochrome *c* interacts with apoptotic protease-activating factor 1 (APAF-1) and procaspase-9 to form the apoptosome complex, which activates caspase-9 (68). Activated caspase-9 subsequently triggers the activation of downstream effector caspases, ultimately leading to apoptosis.

The hypothesis that apoptosis is involved in I/R was largely based on findings that loss or overexpression of apoptotic regulators such as BCL-2 altered the response to I/R injury and that elements of the apoptotic signaling pathway are altered during I/R. It should be noted that recent data suggest an overlap in the regulation of apoptosis and necroptosis, which could account for many of these findings. Studies have shown that an increase in the levels of anti–cell-death proteins such as BCL-2 through genetic modifications reduces tissue damage (69, 70). Although cardiomyocyte-specific BCL-2 overexpression decreased overall cell death by approximately 20%, the reduction in apoptotic cell death was only 3% (70). These data suggest that BCL-2 overexpression reduces not only apoptotic cell death but also necrotic cell death (70, 71). Several studies indicate that these anti–cell-death proteins may have broader protective effects beyond apoptosis, as they can also mitigate other forms of cell death, such as necrosis and autophagy (70, 72–74). It has been shown that inhibiting caspases significantly reduces tissue damage (66, 75, 76). Caspases, particularly caspase-9, appear to be activated during both ischemia and reperfusion, suggesting their involvement in the cell-death process (76).

Pyroptosis is a form of programmed cell death mediated by gasdermin proteins (77, 78) and executed by the activation of inflammatory caspases, particularly caspase-1 and caspase-11 (79, 80). It also involves the assembly of the inflammasome NOD-like receptor 3 (NLRP3), a component that can bind to the OMM (81). This binding facilitates translocation of oxidized mitochondrial DNA (mtDNA) to the cytosol and triggers activation of caspase-1 (81, 82). This process results in release of proinflammatory cytokines, such as IL-1β and IL-18, and induces a robust inflammatory response (83). A role for gasdermins in I/R was shown by Shi et al., who reported that gasdermin D knockout reduced I/R injury (78, 84). It is tempting to speculate that activation of mPTP leads to release of mtDNA, which in turn activates gasdermin-mediated, plasma membrane–mediated death.

### Summary of cell-death pathways

**Figure 4:**
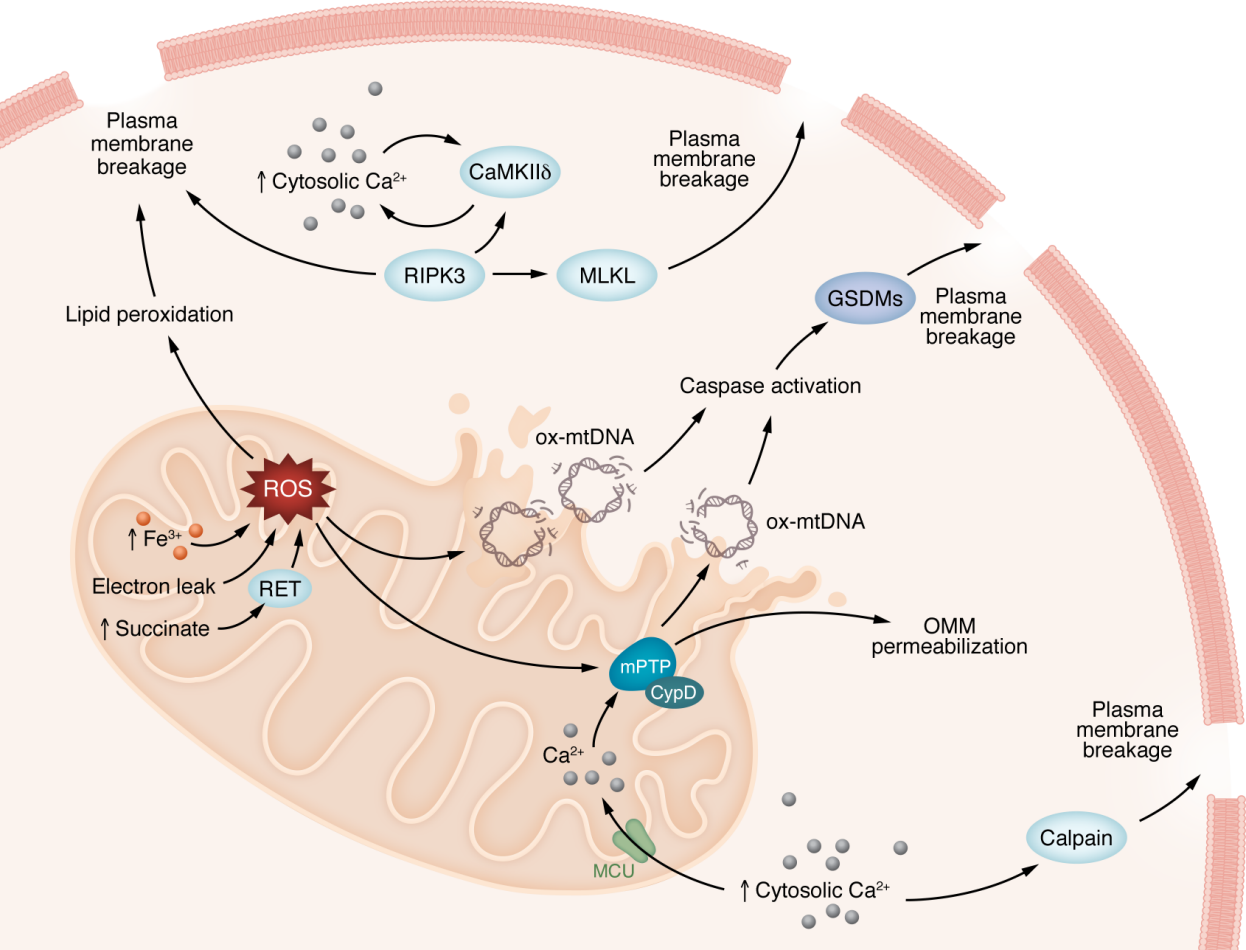
Major contributors of cardiac I/R injury. Myocardial injury during I/R is triggered by multiple cell-death pathways, all of which contribute to the final infarct size. During ischemia, there is an increase in cytosolic Ca^2+^; this leads to the activation of calpains, which can trigger plasma membrane rupture, CaMKII, and mitochondrial Ca^2+^ overload through the MCU. Mitochondrial Ca^2+^ overload is one of the triggers of mPTP opening. CypD is a regulator of the mPTP. ROS are generated during I/R injury by a variety of mechanisms, including, but not limited to, increased Fe^3+^ (canonically ferroptosis), electron leak from damage to the ETC, and the accumulation of succinate leading to RET. ROS contribute to mPTP opening and are also implicated in lipid peroxidation, leading to plasma membrane breakage and oxidization of mtDNA (ox-mtDNA) and its leakage into the cytosol. Once in the cytosol, ox-mtDNA can activate caspases, which can either independently cause plasma membrane breakage or activate gasdermins (GSDMs). GSDMs form pores in the plasma membrane. RIPK3 is also activated, leading to MLKL activation, which forms pores in the plasma membrane.

Many additional cell-death pathways, including autophagy and parthanatos, have been described and are reviewed elsewhere (8). Substantial crosstalk occurs between the different cell death pathways and stressors such as Ca^2+^ and ROS during I/R. Further studies on the connections between these pathways could provide a clearer understanding of the physiological changes occurring in the pathogenesis of I/R. This could explain why some therapies targeting only one pathway have been ineffective. Figure 4 shows how multiple cell-death pathways might interact to cause cell death.

## Cardioprotective strategies

### Pre- and postconditioning

Subjecting hearts to brief periods (typically 5 minutes) of ischemia followed by brief periods of reperfusion before a sustained ischemic insult reduced myocardial infarct size and preserved cardiac function (85). This technique, termed *preconditioning*, also reduced generation of lactate (86, 87), attenuated acidosis (87, 88), slowed the rate of ATP breakdown (86, 88), and diminished arrhythmias during reperfusion (89, 90). Preconditioning showed a protective effect in patients who were undergoing coronary artery bypass surgery (91). Mechanistic studies have reported the involvement of signaling pathways, such as endothelial NOS (eNOS) (92), PKC (93, 94), ERK (95), and inhibition of glycogen synthases kinase (GSK-3β) (92, 96).

As preconditioning needs to be done prior to ischemia, it is of limited clinical benefit. However, an alternative technique known as *postconditioning* has been described (97). This approach involves subjecting the heart to several cycles of sublethal ischemia and reperfusion following the start of reperfusion. Mechanistically, postconditioning activates many of the same signaling kinases as preconditioning. Notably, studies have shown that the protective effects of postconditioning involve adenosine receptors (98, 99). More details on the mechanism of postconditioning are covered in previous reviews (10, 100).

Based on the signaling pathways activated by pre- and postconditioning, a number of studies demonstrated that pharmacological activation of these pathways is cardioprotective. Many different therapeutic strategies to reduce I/R injury have been identified (101), including antiinflammatory compounds (102–104), antioxidants (105, 106), adenosine (107, 108), erythropoietin (109), metabolic modulators (110, 111), atrial natriuretic peptide, and NO. Animal studies have shown that increasing NO levels can reduce infarct size, suggesting its potential for therapeutic use in treating I/R injury (112, 113). Interestingly, the effects of NO on I/R injury appear to be influenced by biological sex (114), with evidence indicating sex differences in the activation of the Ca^2+^-dependent neuronal NOS (nNOS) and eNOS and S-nitrosylation patterns, which are more prevalent in females (114–116).

### Paracrine-mediated protection

Data suggest that some of the conditioning protocols, including remote conditioning, are mediated by paracrine mechanisms involving the release of biologically active signaling molecules and proteins. Stem cell therapy has also been shown to provide protection via similar paracrine signaling mechanisms. It is thought that release of signaling molecules can activate some of the same cardioprotective signaling pathways that are activated by pre- and postconditioning. Exosomes and miRNAs are among the signaling molecules involved in this protective signaling. While some miRNAs have been shown to be harmful by promoting I/R (117), they could potentially be blocked with antagomirs (synthetic RNA duplexes that mimic the endogenous functions of specific miRNAs). Conversely, other miRNAs might be beneficial, as they mitigate damage and could be delivered using nanoparticles or vesicles to reduce harm (118, 119). However, clinical translation of these therapies will require better administration protocols, cell-specific delivery, and additional prognostic models.

### Targeting changes occurring during I/R

As discussed above, a number of ionic, energetic, metabolic, and signaling changes occur during ischemia and reperfusion that can activate cell-death pathways. A number of cardioprotective approaches to reduce the triggers of mPTP (Ca^2+^ and ROS) have been tried.

#### Increased cytosolic Ca^2+^.

Inhibition of plasma membrane Na^+^/H^+^ exchange (NHE) was shown to reduce the ischemic rise in Na^+^ and Ca^2+^ (120) if the inhibitors were given prior to ischemia. However, as the increase in cytosolic Ca^2+^ occurs during ischemia, cardioprotective approaches next focused on pathways downstream of cytosolic Ca^2+^ in an attempt to understand the mechanism by which an increase in cytosolic Ca^2+^ initiates cell death. There are several hypotheses regarding the potential mechanisms by which an increase in cell Ca^2+^ might activate cell death. These include (i) entering the mitochondria to active mPTP; (ii) activating CaMK signaling pathways that initiate death signaling; (iii) activating calpain or similar Ca^2+^-activated proteases; and (iv) initiating sarcoplasmic reticulum (SR) Ca^2+^ overload and Ca^2+^ oscillations, depleting cell ATP.

#### Mitochondrial Ca^2+^ overload.

As the MCU is the main mechanism of Ca^2+^ entry into mitochondria (121, 122), it has been proposed that inhibiting the MCU might reduce mPTP opening (123). Mice with MCU deletion were developed to test this, and their susceptibility to I/R injury tested. In one study, MCU was knocked out at birth, and although isolated mitochondria did not take up Ca^2+^ and undergo mPTP opening, hearts from the germline MCU-knockout mice were not protected from I/R injury (124). In contrast, adult mice with a cardiomyocyte-specific inducible deletion of MCU showed protection from I/R injury (125, 126). Although the reasons for the discrepancy in I/R injury in different MCU-knockout models remain unclear, it has been proposed that germline loss of MCU before or at birth results in compensatory adaptations in the germline MCU-knockout mouse that counteract the protection (127). However, a recent study measuring mitochondrial Ca^2+^ in a Langendorff-perfused heart model showed that an increase in mitochondrial Ca^2+^ occurs during ischemia, therefore limiting the ability to intervene on reperfusion. Moreover, much of the ischemic increase in mitochondrial Ca^2+^ is independent of the MCU (128), raising questions about whether the protection observed with MCU deletion is due to a reduction in mitochondrial free Ca^2+^. Furthermore, in an in vitro model of I/R, it was found that germline loss of the MCU attenuates the rise in mitochondrial Ca^2+^ during simulated ischemia but does not reduce cell death (129). Similarly, in the same in vitro model, acute knockout of the MCU did not alter mitochondrial Ca^2+^ or cell death during I/R (129, 130). Taken together, these data suggest that inhibition of the MCU is unlikely to be a useful target for reducing mitochondrial Ca^2+^ during I/R. However, a number of studies have demonstrated protection when isolated rat hearts were perfused with ruthenium red, which interferes with intracellular Ca^2+^ flux in cardiac muscle (131–135). As discussed in these studies, the purity of ruthenium red is variable, and it has many off-target effects (136). Ru360 was purified from ruthenium red and identified as the active component in MCU inhibition. Studies in perfused rat hearts using Ru360 have demonstrated improved heart function recovery after ischemia when Ru360 was administered prior to ischemia (132, 135). In another study Ru360 was shown to reduce infarct size; Ru360 was injected i.p. into the mouse 30 minutes prior to in vivo LAD (left anterior descending) artery occlusion for 30 minutes, followed by 2 hours of reperfusion (137). These studies suggest that pharmacological inhibition of the MCU is cardioprotective. It will be interesting to examine whether Ru360 reduces the rise in mitochondrial Ca^2+^ during I/R. Mitochondrial Ca^2+^ efflux pathways have also been studied as therapeutic targets to reduce I/R injury. Specifically, the use of conditional transgenesis to overexpress cardiac mitochondrial Na/Ca/Li exchanger (NCLX) augmented mitochondrial Ca^2+^ clearance, prevented mPTP, and protected against ischemia-induced cardiomyocyte necrosis and heart failure in mice (138). However, there are also data suggesting that during ischemia, NCLX can operate to transport Ca^2+^ into the mitochondria (130, 139). Further studies are needed to address these discrepancies.

#### Ca^2+^ activation of CaMKII.

Downstream mediators of cell death, such as CaMKII, also have been targeted in preclinical experiments to reduce I/R injury. Inhibitors of CaMKII such as KN-93 or the CaMKII inhibitory peptide (AIP) have been shown to reduce infarct size by decreasing necrosis and apoptosis (140). As CaMKII can also be activated by ROS, reducing ROS production during I/R reduces CaMKII’s contribution to I/R injury (141). In addition, it has been shown that modifying methionine 281 and 282 in the CaMKIIδ sequence in human induced pluripotent stem cells (iPSCs) and in humanized mice leads to a reduction in I/R injury and confers cardioprotection to the heart against the damage caused by ROS during reperfusion. In this approach, the edited CaMKIIδ was efficiently delivered by myotropic adeno-associated virus directly to the heart before the start of I/R (142).

#### Ca^2+^ activation of proteases.

Ca^2+^ overload upon reperfusion leads to the activation of Ca^2+^-dependent proteases, such as calpains, which cleave proteins, leading to plasma membrane breakage (143, 144). Pharmacological inhibition of calpains upon reperfusion has shown to be protective in multiple animal models (145, 146). The observed protection may be due to the reduction in structural damage caused by increased calpain activity upon reperfusion (143).

#### Sarcoplasmic reticulum (SR) Ca^2+^ overload.

As reviewed elsewhere (147, 148), oscillations in cytosolic Ca^2+^ have been shown to occur on reperfusion, contributing to I/R injury and death. These oscillations in cytosolic Ca^2+^ are primarily due to oscillation in SR Ca^2+^ uptake and release and dysregulation of the sarcoplasmic/endoplasmic reticulum Ca^2+^ ATPase (SERCA), and the ryanodine receptor (RyR2) can contribute to these oscillations.

#### Increased ROS.

As discussed, restoring oxygen on reperfusion restores the activity of electron transport, which, due to damage during ischemia, exhibits increased electron leak, generating ROS. This increase in ROS contributes to tissue damage by activation of the mPTP (149, 150). Furthermore, overproduction of ROS on reperfusion leads to peroxidation of membrane lipids (151) and damages ETC proteins and mtDNA (152). One method to target this increase in ROS involves administering mitochondria-targeted antioxidants at reperfusion, such as mitoTEMPO. MitoTEMPO is a synthetic scavenger of superoxide and alkyl radicals composed of the lipophilic cation triphenylphosphonium (TPP^+^) and piperidine nitroxide (TEMPO) (153). TPP^+^ allows mitoTEMPO to penetrate cell membranes and accumulate within the mitochondria, thereby selectively targeting ROS in the mitochondrial matrix. MitoTEMPO has been shown to be cardioprotective in models of I/R injury (154, 155). Another antioxidant tested for myocardial I/R injury is resveratrol, which scavenges ROS, including superoxide and peroxynitrite (156). Studies show that resveratrol is cardioprotective in preclinical models and humans (157). Other antioxidant therapies tested against I/R injury have been reviewed elsewhere (158).

Reverse electron transport (RET) driven by an increase in succinate generated during ischemia is another important source of ROS during reperfusion, and blocking RET is cardioprotective (159). Succinate accumulates during ischemia, and upon reperfusion succinate is rapidly oxidized by succinate dehydrogenase, leading to RET through complex I, generating ROS (160, 161).

Monoamine oxidases (MAOs) are another source of ROS due to the catabolism of amines (162). MAOs are localized in the OMM and generate H_2_O_2_ during oxidative breakdown of neurotransmitters such as serotonin and norepinephrine. Experiments in rabbits and mice have shown that deletion or inhibition of MAOs resulted in a reduction in infarct size (163). Interestingly, deleting MAO-B in mouse cardiomyocytes reduced infarct size in male mice, whereas female mice were unaffected (164).

## Clinical trials

For patients presenting with an ST-elevation MI (STEMI), acute ischemia has already occurred; however, there is a window of opportunity before percutaneous coronary intervention (PCI) or thrombolysis when pharmacologic intervention can be administered to reduce lethal reperfusion injury. An important issue here is that administering drugs at the beginning of reperfusion will not reverse any damage or changes occurring during ischemia (e.g., an increase in mitochondrial Ca^2+^) that trigger cell death during reperfusion. The clinical trials discussed below are summarized in Table 1.

### Targeting Ca^2+^ overload

Ca^2+^ overload during ischemia can occur as a result of increased Ca^2+^ influx, and studies have shown that administering NHE inhibitors prior to ischemia attenuated the increase in cytosolic Ca^2+^ and were cardioprotective (120). In a large randomized clinical trial, patients undergoing high-risk PCIs were administered cariporide, a selective NHE inhibitor, or a placebo before PCI (165). Administering NHE inhibitors prior to the PCI, as compared with the placebo, did not alter the infarct size or risk of death. These results were similar to those of another clinical trial using the NHE inhibitor eniporide (166). The reasons that NHE inhibitors delivered on reperfusion did not protect against I/R injury have been extensively reviewed, and the results are consistent with the preclinical data, which show that Na^+^ and Ca^2+^ levels increase during ischemia (167). As the NHE inhibitor was administered after the ischemic event, it would not be able to block the increase in cytosolic Na^+^ and Ca^2+^ that occurs during ischemia and therefore would not prevent Ca^2+^-mediated initiation of necrotic cell death. Interestingly, a post hoc analysis of the study found that cariporide was beneficial to patients undergoing coronary artery bypass grafting (CABG) when the drug was administered prior to the ischemic period during surgery (168). This was tested in the EXPEDITION trial, a follow-up clinical trial in which patients undergoing CABG were administered a higher dose of cariporide prior to the procedure (169). As administering cariporide reduced nonfatal MI after CABG but increased the incidence of adverse cerebrovascular events, widespread clinical implementation is unlikely. As demonstrated by the EXPEDITION trial, in order for NHE inhibitors to be protective in humans, they must be administered prior to the start of the ischemic period.

### Targeting mPTP

Inhibiting mPTP opening has been reported to prevent necrotic I/R injury. As the identity of the mPTP is unknown, therapeutic strategies have focused on targeting regulators of the pore. CypD, a regulator of mPTP activation, has been identified as an activator of the pore. Initially, a small pilot trial was conducted in which patients presenting with acute STEMI received either CsA or a saline infusion prior to PCI (170). This study found a 44% reduction in infarct size 5 days after PCI in patients who received CsA compared with the control. This reduction in infarct size remained after 6 months and was associated with reduced cardiac remodeling (171). However, multiple larger follow-up clinical trials found CsA not to be protective as compared with the placebo (172, 173). A possible explanation for the observed lack of protection is that CsA does not directly target the mPTP. It only targets its activator CypD, and preclinical studies have shown that with high levels of activators, the mPTP can still open in the presence of CsA (174–176). Furthermore, CsA has off-target effects such as inhibiting calcineurin phosphatase that might contribute to its lack of protection (177). Another explanation is that there are multiple cell-death pathways and that inhibition of only one (necrosis, via mPTP) is not sufficient (8).

The mitochondrial translocator protein TSPO is another regulator of the mPTP (178, 179). 3,5-seco-4-nor-cholestan-5-one oxime-3-ol (TRO40303) was found to inhibit TSPO by binding to its cholesterol-binding site and was shown to be cardioprotective (178). TRO40303 appears to inhibit mPTP by a different mechanism compared with CsA (178). When evaluated in a phase IIa clinical trial, TRO40303 administered during PCI was found not to alter infarct size as compared with placebo, and it increased the rate of cardiac events (180).

### Targeting ROS

In preclinical studies, MTP-131 (SS-31, Elamipretide, Bendavia), a mitochondria-targeted compound that leads to reduced ROS generation, was found to be protective in animal models of I/R injury (181–183). SS-31 binds to and preserves the integrity of cardiolipin, a phospholipid present in the IMM. This interaction has been shown to optimize electron transport, reduce ROS generation, and improve myocyte survival during reperfusion (183–185). However, when MTP-131 was administered to patients prior to PCI in the EMBRACE STEMI clinical trial, there was no change in infarct size as compared with the placebo (186). Interestingly, when MTP-131 was administered to a small number of patients undergoing angioplasty of the renal artery, it was found to be protective, attenuating the development of renal hypoxia (187). The differences in protection between the two studies are not clear, and further research is needed.

Another strategy to reduce the generation of ROS during reperfusion is administration of mitochondria-targeted antioxidants, such as MitoQ (mitoquinol mesylate). In rats, administering MitoQ prior to ischemia reduced reperfusion injury (188–190). While this strategy has not been tested for I/R in humans, an ongoing clinical trial (NCT05410873) is recruiting patients to determine whether administering MitoQ is beneficial in patients with dilated cardiomyopathy.

### Targeting nitric oxide

Increasing NO levels upon reperfusion in animal studies was cardioprotective. Clinical trials explored NO donors and nitrite to mitigate I/R injury, but the results have been inconsistent. Initially, patients presenting with acute MI were infused with isosorbide dinitrate (191, 192). While no reduction in infarct size was found in either study in the overall cohort, there were differences in infarct size depending on the severity and type of MI. More recently, patients were infused with sodium nitrite prior to administration of reperfusion therapy (193, 194); however, there was no reduction in infarct size. Similar results were also found when inhaled NO was used prior to PCI in humans, with no reduction in infarct size (195). Another trial looked at directly administering sodium nitrite to patients with known inducible myocardial ischemia (196). Using flow-mediated dilation as a marker for recovery after ischemia, it was found that pretreating patients with sodium nitrite resulted in better recovery after ischemia.

## Conclusion

A number of strategies have been shown to provide cardioprotection in animal studies, but these approaches have not been protective in clinical trials. The reasons have been discussed previously (134, 197, 198), but a few are worth reviewing again here. There is a narrow window in which cardioprotection can be applied. If the infarct size is small, it will be difficult to see an improvement. Preclinical studies are also done on young, healthy animals, and the comorbidities present in patients may alter the cardioprotective response. Also, most patients who undergo an MI are typically on a number of other drugs, some of which might alter their response to cardioprotective drugs.

One other limitation in cardioprotective studies is the focus on single targets or pathways. As discussed above, there are a number of cell-death pathways; however, there appears to be crosstalk among these pathways. In I/R, multiple pathways (or parts of a pathway) might be activated and contribute to cell death. A cocktail approach that inhibits multiple pathways should be considered. In line with this, published studies have shown that the use of both necrostatin and CsA has an additive effect in reducing cell death. Similarly, the combination of CsA and ferrostatin has also been shown to reduce infarct size to a greater extent than either drug alone.

## Figures and Tables

**Table 1:**
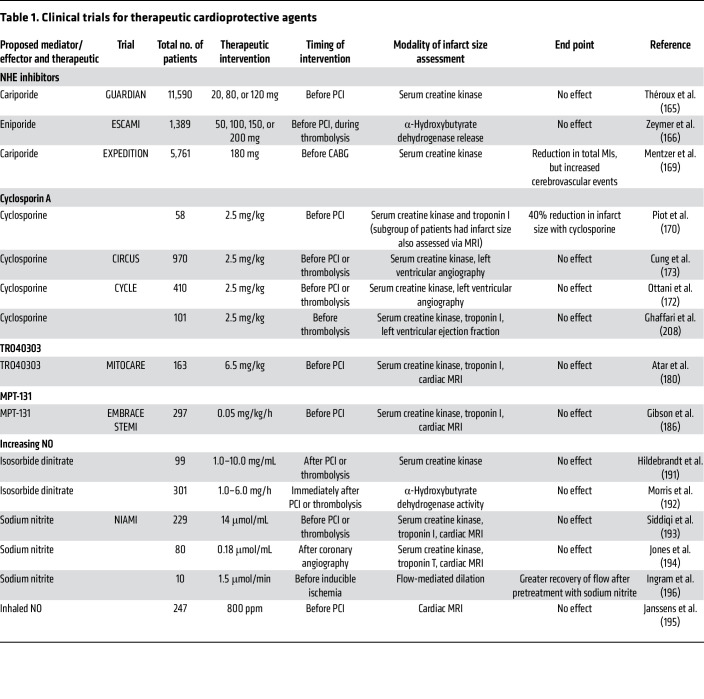
Clinical trials for therapeutic cardioprotective agents

## Version 1 01/02/2025

Electronic publication
